# Emergence of Caenorhabditis elegans as a Model Organism for Dissecting the Gut–Brain Axis

**DOI:** 10.1128/mSystems.00755-21

**Published:** 2021-08-24

**Authors:** Lizett Ortiz de Ora, Elizabeth N. Bess

**Affiliations:** a Department of Chemistry, University of California, Irvine, California, USA; b Department of Molecular Biology and Biochemistry, University of California, Irvine, California, USA

**Keywords:** *C. elegans*, gut microbiome, gut–brain axis, gnotobiotics, neurosignals, neurodegenerative diseases

## Abstract

Accumulating evidence links the gut microbiome to neuronal functions in the brain. Given the increasing prevalence of brain disorders, there is a critical need to understand how gut microbes impact neuronal functions so that targeted therapeutic interventions can be developed. In this commentary, we discuss what makes the nematode Caenorhabditis elegans a valuable model for dissecting the molecular basis of gut microbiome-brain interactions. With a fully mapped neuronal circuitry, C. elegans is an effective model for studying signaling of the nervous system in a context that bears translational relevance to human disease. We highlight C. elegans as a potent but underexploited tool to interrogate the influence of the bacterial variable on the complex equation of the nervous system. We envision that routine use of gnotobiotic C. elegans to examine the gut–brain axis will be an enabling technology for the development of novel therapeutic interventions for brain diseases.

## COMMENTARY

Although the bidirectional communication between the human gastrointestinal (GI) tract and the brain has long been recognized, much remains to be discovered about the contribution of gut microbes to the gut–brain axis. Signals arising from both the brain and the gut are crucial in the regulation of gut motility as well as hormone secretion that coordinates hunger and satiety (1). Similarly, signaling molecules that originate within the gut, such as microbial metabolites, may influence the human brain (2). Advances in next-generation sequencing technologies and studies with gnotobiotic mice have revealed associations between the gut microbiota and neurological processes such as neurodevelopment (3), host behavior (3–5), and incidence of neurodegenerative disorders (6, 7). Emerging from these findings is an avenue to prevent, diagnose, and treat diseases of the brain by leveraging the malleable gut microbiome. Yet, for this goal to be achieved, it is crucial to identify and dissect the molecular-level mechanisms behind these associations.

Gnotobiotic mice are often the preferred model organism to study host–microbe interactions due to their anatomic and genetic similarity to humans. While studies in these gnotobiotic animals have yielded critical insights in the field, deciphering molecular-level pathways connecting gut microbiome functions to their impacts on the brain has remained challenging due to the complex physiology of the mammalian host. For instance, 80 to 100 million neurons comprise the murine enteric nervous system that innervates the mammalian gut and communicates with the billions of neurons of the central nervous system (8). These neurons and their functions are incompletely characterized, in part, due to the technical difficulty in monitoring these biological processes in real time. An attractive alternative model organism that may facilitate elucidation of gut bacterial mechanisms modulating neuronal functions is the nematode Caenorhabditis elegans, which has a significantly simpler and fully characterized nervous system. The transparent body and genetic trackability of this nematode enable *in situ* visualization of fluorescently labeled microbes as well as genetically encoded fluorophores to label and track host-produced proteins. These features, combined with a short life span and cost-effective gnotobiology protocols, enable high-throughput experimental approaches that are not possible in mice. Here, we discuss the characteristics that can position C. elegans as a model of choice to decipher molecular-level pathways impacting the gut microbiome–brain axis.

## C. ELEGANS AS A TRACTABLE GNOTOBIOTIC MODEL

C. elegans offers several advantages to model host–microbe interactions. Perhaps the most important of them is that these nematodes naturally engage in interactions with bacteria (9). An important host–predator relationship exists as C. elegans survives on a diet of bacteria. Although C. elegans has a pharyngeal grinder that disrupts most bacterial cells (10), mounting evidence indicates that bacteria that escape the grinder can establish symbiotic relationships with their host by colonizing and proliferating in the digestive tract of adult nematodes. The generation of C. elegans mutant strains with a defective grinder has demonstrated that accumulation of nonpathogenic bacteria in the gut extends the life span of the nematode (10). Additionally, C. elegans requires metabolically active bacteria for its normal development and growth. With at least 83% of the C. elegans proteome sharing homology with human proteins (11), there is accumulating evidence that the impacts of microbes on physiological processes of the nematode can illuminate host–microbiome interactions that translate to the mammalian host (12).

**FIG 1:**
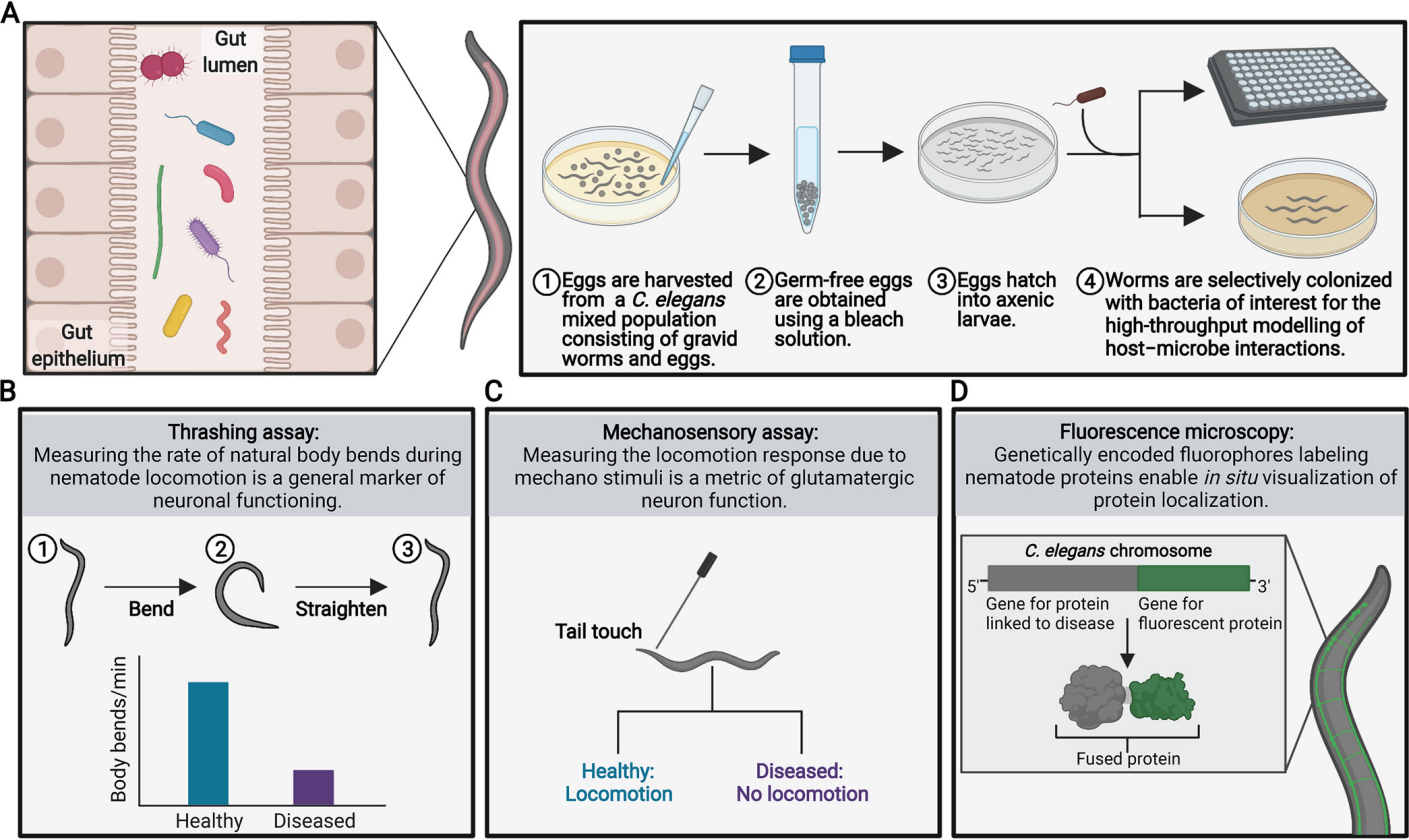
Modeling gut microbiome–brain interactions in the tractable C. elegans gnotobiotic model. (A) The digestive tract of C. elegans can be selectively colonized with bacteria of interest by using a simple and inexpensive four-step protocol. (B to D) The discovery of mechanisms by which gut bacteria can impact neurological processes in C. elegans can benefit from the extensive characterization of phenotypes associated with neuronal functions in this organism. Some of these phenotypes include (B) thrashing rate, (C) mechanosensory response, and (D) expression of fluorescence-labeled proteins in C. elegans transgenic models of disease. Figures created with BioRender.com (agreement number QL22QJZRC2).

A particularly advantageous trait of C. elegans is the simplicity with which host-microbe interactions can be modeled (Fig. 1A). Age-synchronized germ-free populations can be easily obtained by treating C. elegans cultures with bleach. This treatment kills both adult nematodes and their bacterial diet, leaving only germ-free bleach-resistant eggs that can hatch into axenic larvae. Hatched germ-free C. elegans can be selectively colonized with bacteria of interest to interrogate the impact of the gut microbiome on various biological processes of the host.

Due to its millimeter size, the C. elegans nematode is amenable to high-throughput experimentation. Hundreds of animals can be inexpensively studied on petri dishes and in 96-well plates (Fig. 1A). While the bacterial communities that can colonize the C. elegans digestive tract contain a smaller number of taxa relative to humans, this feature enables the comprehensive study of simplified mock communities to understand the foundational mechanisms that govern host–microbe interactions. Remarkably, the cell-fate map of every cell in C. elegans has been described (13); thus, this nematode is uniquely positioned for the precise dissection of host–microbe cellular functions and interactions. Additionally, C. elegans’ transparent body and genetic trackability enable the use of fluorescently labeled bacteria and transgenic nematodes to visualize and juxtapose host–microbe interactions in real time. These characteristics make C. elegans an attractive gnotobiotic model for examining host–microbe interactions.

## C. ELEGANS AS A MODEL FOR THE STUDY OF HOST–MICROBE NEUROSIGNALING

Along the gut–brain axis, information can be transmitted by numerous combinations of hormones and neurotransmitters. In humans, these signaling molecules are commonly associated with the brain; however, they are also prevalent in the GI tract. For instance, over 90% of serotonin (14) and nearly half of all dopamine (15) in the human body are produced in the gut. Remarkably, a growing body of evidence suggests that gut microbes play a pivotal role in modulating the levels of these and other neurosignaling molecules in the GI tract. The production of hormones and neurotransmitters by enteroendocrine cells is known to be stimulated by the gut microbiome (16). Additionally, gut bacteria can directly sense, synthesize, and degrade neuroendocrine signals (5, 16, 17), yet the impacts of these metabolic activities *in vivo* remain incompletely characterized. As in humans, communication between the gut and brain via neuroendocrine signals occurs in C. elegans. Although more research is required to completely characterize the metabolome of the nematode’s intestine, the existence of receptors for dopamine, serotonin, and other neuroendocrine signals in the digestive tract that respond to bacterial cues suggests that C. elegans is a well-suited platform for identifying and characterizing the gut microbiota’s neuroactive potential. With a fully mapped neuronal circuitry (18) as well as extensive characterization of phenotypes associated with neurosignals (Fig. 1B to D), this nematode enables the study of neuronal communication to an extent not yet possible in any other animal species.

The nervous system of an adult C. elegans hermaphrodite consists of only 302 neurons that innervate its body. The synaptic connections and functions of every neuron in C. elegans have been characterized (18). Although the mammalian nervous system consists of billions of neurons with incompletely understood synaptic connections, the molecular and cellular functions of neurons are highly conserved between C. elegans and mammals (11). Additionally, there exist multiple genetically engineered C. elegans strains in which key proteins in neuronal circuitry are fluorescently labeled; novel mutant strains can also be readily developed. The exceptional genetic and phenotypic characterization of C. elegans’ neurological processes as well as the vast genetic toolbox for this organism poises this model for use in precisely mapping the interactions between microbes and neuronal functions.

## MODELING HOST–MICROBE INTERACTIONS IN NEURODEGENERATIVE DISEASES IN C. ELEGANS

One of the barriers when studying host–microbe interactions in neurodegenerative diseases is that these disorders tend to appear in senescence in humans and in mouse models. Thus, studying such diseases in mice can be challenging, time-consuming, and expensive. In contrast, several transgenic C. elegans models have been generated to recapitulate certain aspects of human neurodegenerative diseases within the short 20-day life span of the nematode (19–21). This advantage has enabled the rapid screening of C. elegans mutant strains to identify nematode genes associated with aging processes and neurodegenerative phenotypes as well as high-throughput testing of chemicals for their therapeutic potential. While the impact of host aging processes on the native gut microbiome in C. elegans remains an open question, gnotobiotic C. elegans has proved to be a valuable model for deciphering specific bacterial processes that affect longevity (10). Nonetheless, C. elegans remains underexploited for understanding the microbial factors that impact neurodegeneration.

## LESSONS FROM C. ELEGANS TRANSGENIC MODELS TO UNDERSTAND THE ROLE OF THE GUT MICROBIOME IN PD

The few reports using C. elegans as a model organism to interrogate the role of the gut microbiome in neurodegenerative diseases have been focused, almost exclusively, on Parkinson’s disease (PD) (22–24). In humans, a hallmark of PD is the aberrant aggregation of the protein α-synuclein. Accumulation of α-synuclein aggregates in the brain leads to neuronal death, ultimately provoking motor dysfunction. Recent evidence suggests that the gut microbiome is implicated in the initiation of this pathogenic aggregation (6). C. elegans models of PD have been useful for gaining insights into how gut bacteria can ameliorate (24) or exacerbate (22, 23) α-synuclein aggregation. Notably, a bacterial protein found to induce α-synuclein aggregation in C. elegans has been shown to cause a similar effect in the mammalian gut, demonstrating the translational potential of this nematode to mammalian biological processes (23). Yet, the mechanisms by which the gut microbiota modulate α-synuclein aggregation remain to be deciphered. These mechanisms are likely complex and involve multiple microbial and host factors, including an immune response (6, 23, 25). C. elegans lacks an adaptive immune system, which reduces a layer of complexity in the pathogenic α-synuclein aggregation mechanism without altering its basic principles. This simplification may facilitate distilling the gut microbiome’s impact on α-synuclein aggregation from other host variables.

Thus, C. elegans represents a platform that balances the simplicity of an invertebrate animal model with the power of high-throughput experimental approaches for rapid assessment of features that characterize neurodegenerative disease. This balance makes the nematode an ideal starting model for revealing specific bacterial species, genes, and metabolites of the gut microbiome that modulate PD neurodegeneration. Discoveries in C. elegans may reveal gut microbiome biomarkers for predicting an individual’s predisposition to PD as well as bacterial targets for novel intervention strategies.

In addition to the promise of using C. elegans to understand the role of gut bacteria in PD, there are several other neurodegenerative disorders that also have been associated with the gut microbiome and for which a C. elegans model exists. These models of neurodegeneration include, but are not limited to, Alzheimer’s disease, amyotrophic lateral sclerosis (ALS), frontotemporal dementia, and Huntington’s disease, all of which are considered to be impacted by gut microbial factors that remain largely unknown (7). Because of C. elegans’ legacy in neurobiology research in addition to the several established behavioral assays associated with models of neurodegenerative disease, this nematode is well suited for advancing discoveries of the underlying mechanisms linking the gut microbiome to neurodegenerative disease. We envision that proliferative use of this simple but elegant model organism will complement gnotobiotic mouse models to unravel the mysteries of the gut–brain axis.
